# Enhancing Tamoxifen Therapy with α-Mangostin: Synergistic Antiproliferative Effects on Breast Cancer Cells and Potential Reduced Endometrial Impact

**DOI:** 10.3390/ph16111576

**Published:** 2023-11-08

**Authors:** Rafael Vargas-Castro, Rocío García-Becerra, Lorenza Díaz, Euclides Avila, David Ordaz-Rosado, Samantha V. Bernadez-Vallejo, Saúl Cano-Colín, Javier Camacho, Fernando Larrea, Janice García-Quiroz

**Affiliations:** 1 Departamento de Biología de la Reproducción Dr. Carlos Gual Castro, Instituto Nacional de Ciencias Médicas y Nutrición Salvador Zubirán, Ciudad de Mexico 14080, Mexico; rrvvcc14@gmail.com (R.V.-C.); lorenza.diazn@incmnsz.mx (L.D.); euclides.avilac@incmnsz.mx (E.A.); david.ordazr@incmnsz.mx (D.O.-R.); samantha.bernadezv@incmnsz.mx (S.V.B.-V.); fernando.larreag@incmnsz.mx (F.L.); 2 Departamento de Biología Molecular y Biotecnología, Instituto de Investigaciones Biomédicas, Universidad Nacional Autónoma de México, Ciudad de Mexico 04510, Mexico; rocio.garciab@biomedicas.unam.mx (R.G.-B.); saulcc@unam.mx (S.C.-C.); 3 Programa de Investigación de Cáncer de Mama, Instituto de Investigaciones Biomédicas, Universidad Nacional Autónoma de México, Ciudad de Mexico 04510, Mexico; 4 Departamento de Farmacología, Centro de Investigación y de Estudios Avanzados del I.P.N., Ciudad de Mexico 07360, Mexico; fcamacho@cinvestav.mx

**Keywords:** α-mangostin, breast cancer, tamoxifen, combination index, synergism, endometrium cells, KCNH1

## Abstract

Breast cancer is the most prevalent neoplasia among women worldwide. For the estrogen receptor-positive (ER+) phenotype, tamoxifen is the standard hormonal therapy; however, it carries the risk of promoting endometrial carcinoma. Hence, we aimed to evaluate the antiproliferative effect of the phytochemical α-mangostin (AM) as a co-adjuvant alongside tamoxifen on breast cancer cells to improve its efficacy while reducing its adverse effects on endometrium. For this, ER+ breast cancer cells (MCF-7 and T-47D) and endometrial cells (N30) were treated with AM, 4-hydroxytamoxifen (4-OH-TMX), and their combination. Cell proliferation was evaluated using sulforhodamine B assay, and the pharmacological interaction was determined through the combination index and the dose reduction index calculation. The genes *KCNH1*, *CCDN1, MKI67*, and *BIRC5* were amplified by real-time PCR as indicators of oncogenesis, cell cycle progression, cell proliferation, and apoptosis, respectively. Additionally, genes involved in ER signaling were analyzed. In breast cancer cells, the combination of AM with 4-OH-TMX showed a synergistic antiproliferative effect and favorable dose reduction. AM and 4-OH-TMX decreased *KCNH1*, *CCND1*, and *BIRC5* gene expression. In endometrial cells, AM decreased *MKI-67* gene expression, while it reverted the 4-OH-TMX-dependent *CCND1* upregulation. This study establishes the benefits of incorporating AM as a co-adjuvant for first-line ER+ breast cancer therapy.

## 1. Introduction

Breast cancer is the most frequently diagnosed neoplasia and the leading cause of cancer death in women worldwide [1]. Considering the molecular expression profile, there are three main subtypes of breast tumors: luminal, human epidermal growth factor receptor type 2 (HER-2)-enriched, and triple-negative [2]. Among these, luminal tumors, typically characterized as estrogen receptor α positive (ER+), make up approximately 75% of diagnosed breast cancers [3]. About half of these tumors also express the progesterone receptor (PR), a gene under the transcriptional control of ER [4]. Considering the breast tumors molecular profile, a personalized therapeutic strategy is established. For ER+ breast tumors, tamoxifen, a selective ER modulator, is the most frequently prescribed anticancer drug for pre-menopausal and post-menopausal women. Additionally, it is employed to prevent hormone-dependent breast cancer in high-risk individuals [5,6]. Unfortunately, while tamoxifen exerts anti-estrogenic effects in breast tissue, it can elicit pro-estrogenic effects in the endometrium, leading to an increased risk for developing endometrial lesions including polyps, hyperplasia, metaplasia, and cancer [7]. Consequently, tamoxifen was categorized as a human carcinogen by the International Agency of Research on Cancer in 1996 [7]. Thus, despite its high effectiveness, the undesirable side effects associated with tamoxifen limit its clinical utility. Therefore, identifying co-adjuvants that help to increase the therapeutic effect of this hormonal therapy, allowing dose reduction, while reducing its adverse effects, represents a promising strategy for managing ER+ breast cancer. One of the main approaches in cancer research involves combining conventional drugs with natural antineoplastic compounds to enhance treatment outcomes and to reduce toxicity. In this regard, different plants and fruits possess bioactive compounds with potential therapeutic applications, whose benefits have been attributed to their phytochemicals [8]. In this context, the fruit, leaves, and bark of the mangosteen, a native tree of Southeast Asia, have been used for a long time as a traditional medicine to address disorders affecting the respiratory and digestive systems, urinary and reproductive organs, as well as skin diseases. Mangosteen benefits have been attributed mainly to its xanthones, the most important phytochemicals contained in this tree [9]. Specifically, α-mangostin (AM) is the most abundant xanthone in the mangosteen fruit’s pericarp, exhibiting a wide range of biological activities, with its antineoplastic effects being particularly outstanding [10]. The antitumoral effects of AM have been widely evaluated in different malignancies [10], including breast cancer [11]. In this neoplasia, the antineoplastic effects of AM have been evaluated per se [11] and in combination with doxorubicin [12] and 5-fluorouracil [13], exhibiting a synergic antineoplastic effect. This suggests that AM could be used as a potent co-adjuvant for conventional cancer therapy. Taking into account the latter, we opted to evaluate AM combination with tamoxifen in ER+ breast cancer cell lines. Our objectives were as follows: (a) To improve the antiproliferative effect of hormonal therapy. (b) To ascertain the pharmacological interaction between the compounds by calculating the combination index and dose reduction index (DRI). This would enable us to discern if the interaction is synergistic, additive, or antagonistic and assess the potential for reducing the dosage of the combined compounds. (c) To understand how these compounds influence the expression of genes associated with oncogenesis, cell cycle progression, proliferation, and ER signaling. (d) To assess whether AM mitigates the side effects of tamoxifen in endometrium, using an immortalized cell line derived from this tissue.

Since tamoxifen requires to be metabolized to 4-hydroxytamoxifen (4-OH-TMX) to gain greater activity [14], we decided to use this metabolite to carry out the combination with AM. Additionally, it is noteworthy that 4-OH-TMX reduces the viability of ER+ breast cancer cell lines even in the absence of estradiol [15].

## 2. Results

### 2.1. AM and 4-OH-TMX Inhibited ER+ Breast Cancer Cells Proliferation in a Concentration-Dependent Manner

**Figure 1:**
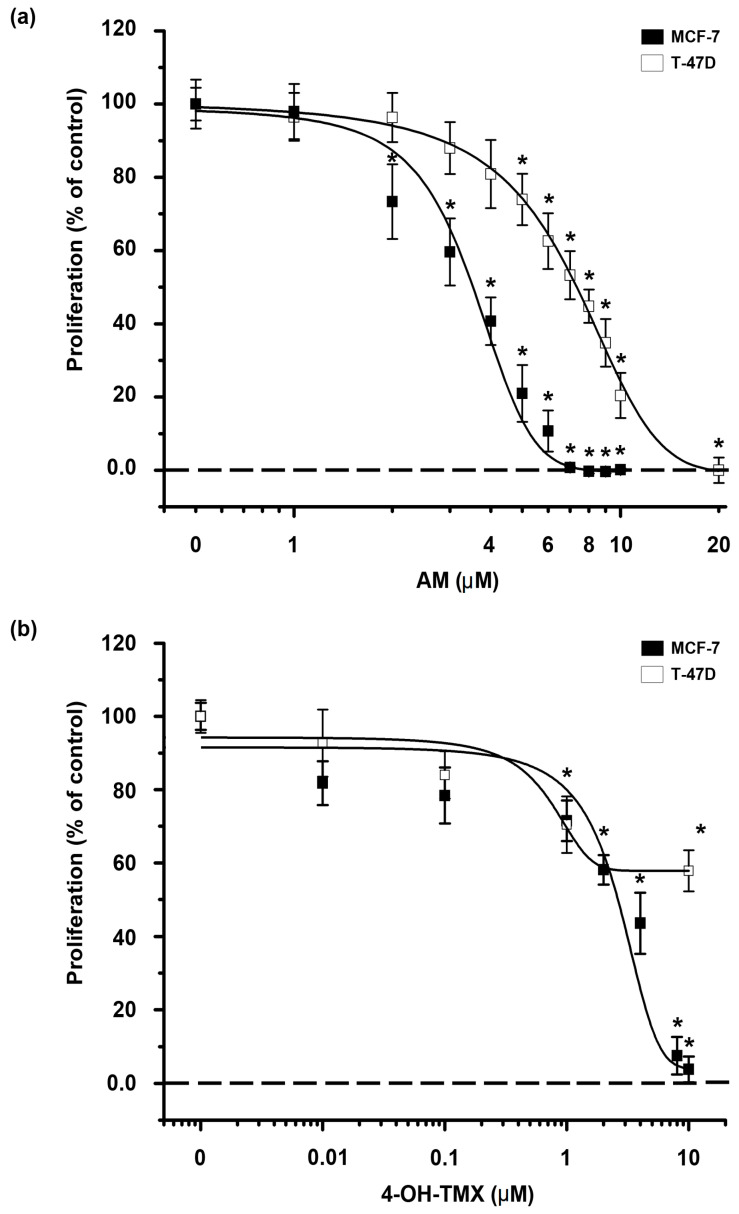
Antiproliferative effect of AM and 4-OH-TMX on MCF-7 and T-47D breast cancer cell lines. The cells were treated with increased concentrations of (**a**) α-mangostin (AM) and (**b**) 4-hydroxy-tamoxifen (4-OH-TMX) for 6 days, followed by the analysis of proliferation by the sulforhodamine B (SRB) assay. As depicted, 4-OH-TMX and AM inhibited MCF-7 and T-47D cell proliferation in a concentration-dependent manner. Results are the mean ± SEM of at least four independent experiments. The data from the vehicle-treated cells were normalized to 100%. * *p* < 0.001 vs. vehicle.

First, we determined the effect of AM and 4-OH-TMX upon the proliferation of the established human ER+ breast cancer cell lines MCF-7 and T-47D. Based on previous studies in breast cancer, the effect of AM on cell proliferation was assessed at concentrations ranging from 1 µM to 20 µM [13,16]. In both breast cancer cell lines, AM significantly reduced cell proliferation in a concentration-dependent manner, as shown in Figure 1a. Notably, AM was able to completely inhibit cell growth of both MCF-7 and T-47D cells at 7 µM and 20 µM, respectively, with MCF-7 cells being more sensitive to this compound than T-47D (Figure 1a).

On the other hand, the effect of 4-OH-TMX on cell proliferation was assessed at concentrations ranging from 0.01 µM to 10 µM in both cell lines, considering previous reports [15]. In MCF-7 cells, 4-OH-TMX significantly inhibited cell proliferation in a concentration-dependent manner, achieving complete inhibition at 10 µM. In contrast, in T-47D cells, 4-OH-TMX significantly inhibited cell proliferation by about 40% at 10 µM (Figure 1b).

To determine the inhibitory concentrations at 20% (IC_20_) and 50% (IC_50_) of AM and 4-OH-TMX (Table 1), we analyzed their respective concentration–response curves. We considered only the minimum and maximum effect of the drugs between the ranges of concentrations evaluated. The IC_50_ values of AM for both cell lines corroborate that the phytochemical is significantly more potent in inhibiting MCF-7 cell proliferation than T-47D cells. Regarding 4-OH-TMX, despite the depicted values, the data related to T-47D IC_50_ values do not reflect a higher sensitivity as compared to MCF-7 cells, given that 100% cell proliferation inhibition was not reached in the former cells (Table 1).

### 2.2. The Combination of AM with 4-OH-TMX Acted Synergistically to Inhibit Cell Growth, Allowing for a Significant Dose Reduction While Maintaining Their Efficacy

**Figure 2:**
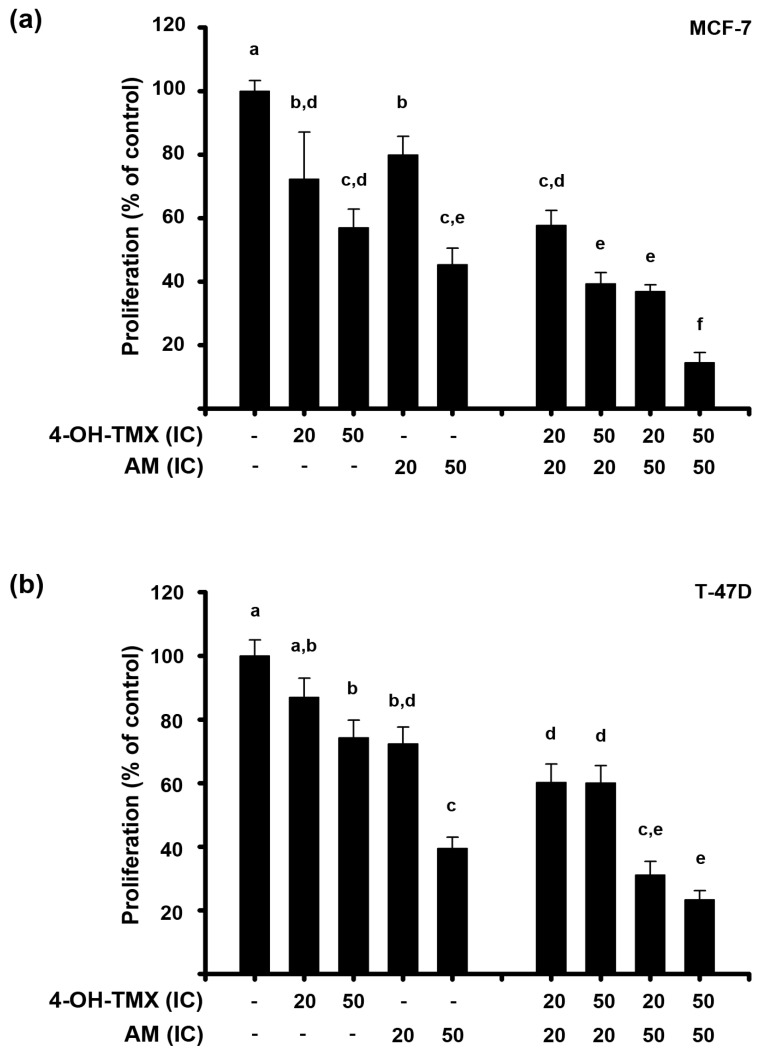
Antiproliferative effect of 4-OH-TMX and AM in combination. (**a**) The antiproliferative effect of 4-OH-tamoxifen (4-OH-TMX) and α-mangostin (AM) was evaluated at their respective inhibitory concentrations at 20% (IC_20_) and 50% (IC_50_) in MCF-7 and (**b**) T-47D cell lines. For further details on inhibitory concentrations, refer to Table 1. After 6 days of treatment, the cell proliferation was evaluated by the sulforhodamine B (SRB) assay. Results are shown as the mean ± SEM of at least five independent experiments. Data from vehicle-treated cells were normalized to 100% and are depicted as the first bars in each graphic. The letters above the bars indicate significant statistical differences (*p* ˂ 0.05) among the treatment groups, assuming that bars lacking a shared letter are considered significantly statistically different. These differences were determined by a one-way analysis of variance, followed by the post-hoc Holm–Sidak method for multiple comparisons.

To evaluate the antiproliferative effects of 4-OH-TMX combined with AM, the following combination schemes were considered (4-OH-TMX:AM): IC_20_:IC_20_, IC_50_:IC_20_, IC_20_:IC_50_, and IC_50_:IC_50_. As shown in Figure 2, combining both drugs reduced breast cancer cell proliferation to a greater extent than each compound alone. The combination scheme of IC_50_:IC_50_ showed the greatest antiproliferative effect, inhibiting cell growth by 83% in MCF-7 cells and around 77% in T-47D cells (Figure 2a,b, respectively). As mentioned above, 4-OH-TMX was not very effective in inhibiting T-47D cell proliferation, but when combined with AM, the antiproliferative effect considerably increased. In this regard, the antiproliferative effect of 4-OH-TMX at its IC_20_ was not significantly different from the control. However, when it was combined with the IC_50_ of AM, the effect was significantly greater than that of each compound alone, and even greater than that elicited by the IC_50_ of 4-OH-TMX per se.

**Figure 3:**
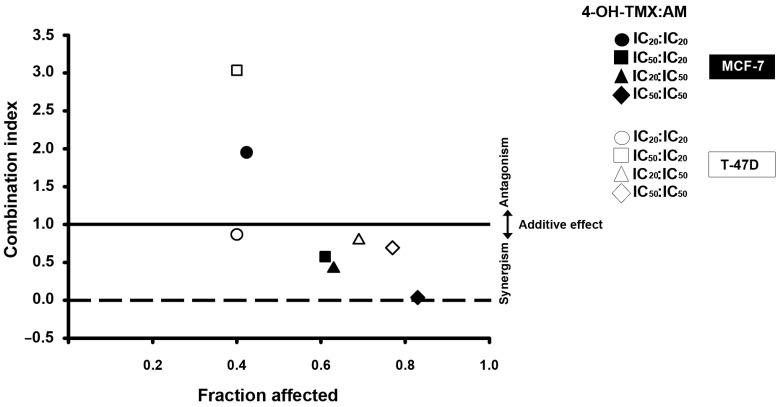
Combination index values as a function of the fraction affected in ER+ breast cancer cells. The combination index and the fraction affected were determined by combining 4−OH−tamoxifen (4−OH−TMX) and α-mangostin (AM) at their respective inhibitory concentrations at 20% (IC_20_) and 50% (IC_50_) in MCF−7 (black symbols) and T−47D (white symbols) cell lines. For further details on inhibitory concentrations, refer to Table 1. Symbols below, on, or above the horizontal line (which indicates a value of 1) represent synergism, addition, or antagonism, respectively. *n* ≥ 5 independent experiments.

To evaluate the nature of the pharmacological interaction between 4-OH-TMX and AM, as well as the potential benefits of their combination, we calculated the combination index. In Figure 3, the combination index is plotted on the *Y*-axis as a function of fraction affected on the *X*-axis. The fraction affected refers to the proportion of cells that are affected or inhibited. This was performed to assess whether there is drug synergism, an additive effect, or antagonism between drug combinations. A combination index value less than one indicates synergism, equal to one suggests an additive effect, while greater than one reflects antagonism [17,18]. In both cell lines, synergism was observed in most combination schemes (Figure 3). To interpret the results, we considered the different levels of synergism and antagonism based on the range of the combination index theorem of Chou-Talalay [17]. In this context, values closer to zero indicate more significant synergism than those closer to one, which can be interpreted as nearly at the additive effect. In MCF-7 cells, the combination of 4-OH-TMX and AM at IC_50_:IC_20_ (black square) and IC_20_:IC_50_ (black triangle) yielded combination indices of 0.574 and 0.415, respectively, demonstrating a synergistic effect. Furthermore, when combined at IC_50_/IC_50_ (black rhombus), the combination index value was 0.037, which suggests very strong synergism. However, the IC_20_:IC_20_ combination (black circle) resulted in a combination index of 1.95, indicating antagonism. In the T-47D cells, the combination of 4-OH-TMX with AM at IC_20_:IC_20_ (white circle), IC_20_:IC_50_ (white triangle), and IC_50_:IC_50_ (white rhombus) yielded combination index values of 0.87, 0.79, and 0.69, indicating slight synergism, moderate synergism, and synergism, respectively. However, when the compounds were combined at IC_50_:IC_20_ (white square), the combination index was 3.04, indicating antagonism. These results suggest a better outcome at higher AM concentrations.

To determine the extent to which the dose of each drug in combination could be reduced, we calculated the DRI values for all combination schemes in both cell lines (Table 2). A DRI value >1, =1, and <1 indicates a favorable, no effect, or negative dose reduction, respectively. A favorable dose reduction refers to how many folds the dose of each drug in combination can be reduced while maintaining the same effect as the dose of the drug alone. The dose reduction may translate into a decreased toxicity in therapeutic applications. Notably, the DRI analysis showed a favorable dose reduction in synergistic combinations, while antagonistic combinations resulted in DRI values below one. Interestingly, the greatest DRI value of 4-OH-TMX and AM in both cell lines was observed with the combination scheme of IC_50_:IC_50_. This highlights that the more significant the synergism, the greater the dose reduction achieved.

### 2.3. The Combination of AM with 4-OH-TMX Enhanced Its Inhibitory Effects upon mRNA Expression of Some Genes Involved in Oncogenesis, Cell Cycle Progression, and Apoptosis in Breast Cancer Cells

To study the mechanisms underlying cell proliferation, we analyzed the effects of the compounds, both individually and in combination, on the gene expression of two cancer-related genes: the oncogenic voltage-gated potassium channel subfamily H member 1 (*KCNH1*) gene and the cyclin D1 gene (*CCND1*).

**Figure 4:**
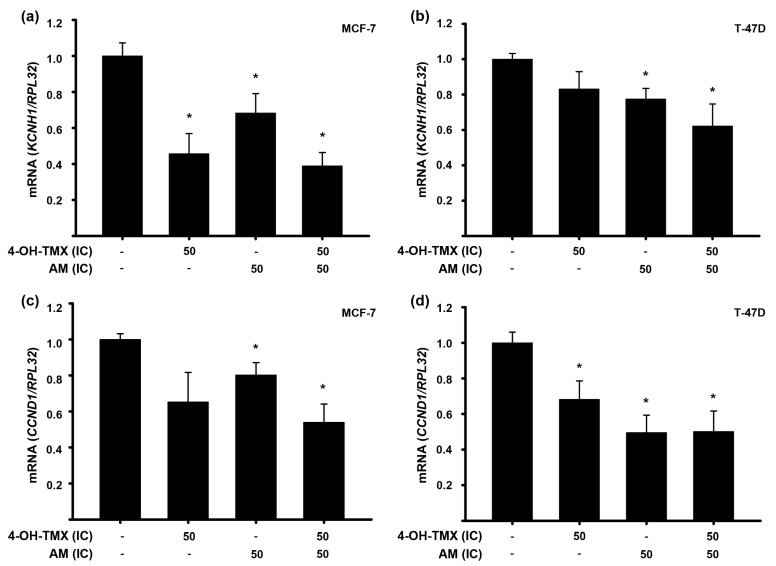
4-OH-TMX and AM decrease *KCNH1* and *CCND1* gene expression in breast cancer cells. MCF-7 (**a**,**c**) and T-47D (**b**,**d**) cell lines were treated with 4-OH-tamoxifen (4-OH-TMX) and α-mangostin (AM), alone and in combination, at their respective inhibitory concentrations at 50% (IC_50_). For further details on inhibitory concentrations, refer to Table 1. The results are shown as the mean ± SEM of relative gene expression of *KCNH1* (**a**,**b**) and *CCND1* (**c**,**d**) after normalizing against the housekeeping gene ribosomal protein (*RPL32*). The data from the treatments were normalized to the vehicle, to which the value of 1 was arbitrarily given.Results from vehicle treatments are represented by the first bars of each graph. * *p* ˂ 0.05 vs. vehicle, *n* ≥ 4 independent experiments.

The *KCNH1* gene encodes the ether-a-go-go 1 (EAG1, Kv10.1) potassium channel, which is overexpressed in various types of cancer, while its inhibition decreases cancer cell proliferation [19]. Our previous studies have demonstrated that AM decreased *KCNH1* gene expression in cervical cancer cells, both in vitro and in vivo [20]. Based on these findings, we aimed to investigate whether this repressive effect could also be observed in breast cancer cells. Therefore, we evaluated the effect of the compounds at their respective IC_50_ values in both cell lines. In MCF-7 cells, the gene expression of *KCNH1* was significantly reduced by both AM and 4-OH-TMX. Combining 4-OH-TMX and AM did not result in additional inhibition (Figure 4a). Regarding T-47D cells, AM significantly inhibited *KCNH1* gene expression, whereas 4-OH-TMX did not exert any effect upon this gene. The compounds’ combination further reduced *KCNH1* gene expression. Although this reduction was not statistically different from the effects of 4-OH-TMX or AM alone, it was significant when compared to the vehicle (Figure 4b).

On the other hand, it is known that the *CCND1* gene, encoding cyclin D1 protein, is crucial in regulating cell cycle progression. Overexpression of this gene is frequently observed in breast cancer and is linked to a positive ER status. Additionally, *CCND1* amplification predicts reduced recurrence-free survival and overall survival in breast cancer patients treated with endocrine therapy [21]. In our study, we found that AM, at its IC_50_ value, significantly inhibited *CCND1* gene expression in both cell lines (Figure 4c,d). However, 4-OH-TMX only exerted this effect in T-47D cells (Figure 4d). The combined treatment of 4-OH-TMX with AM did not further inhibit *CCND1* gene expression in either cell lines (Figure 4c,d).

**Figure 5:**
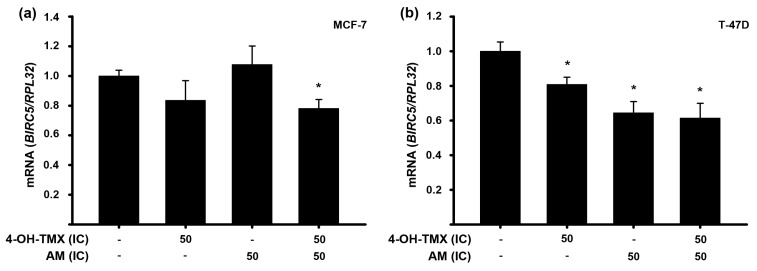
Effect of 4-OH-TMX and AM upon *BIRC5* gene expression in breast cancer cells. MCF-7 (**a**) and T-47D (**b**) cell lines were treated with 4-OH-tamoxifen (4-OH-TMX) or α-mangostin (AM), alone and in combination, at their respective inhibitory concentrations at 50% (IC_50_). For further details on inhibitory concentrations, refer to Table 1. The results are shown as the mean ± SEM of *BIRC5* relative gene expression after normalizing against the housekeeping gene ribosomal protein (*RPL32*). The data from the treatments were normalized to the vehicle, to which the value of 1 was arbitrarily given, and are represented by the first bars of each graphic. * *p* ˂ 0.05 vs. vehicle, *n* ≥ 3 independent experiments.

We also assessed the impact of the treatments on the expression of the *BIRC5* gene, which encodes for survivin. This protein plays a significant role in several cancer-related processes, including cell proliferation, invasiveness, migration, and inhibits apoptosis [22,23]. Regarding the latter, survivin inhibits apoptosis directly or indirectly by interfering with caspase-3, caspase-7, and caspase-9, as well as in a caspase-independent manner [24]. Our results showed that in MCF-7 cells, neither 4-OH-TMX nor AM individually affected the gene expression of *BIRC5*. However, when combined, its expression was significantly inhibited compared to vehicle-treated cells (Figure 5a). In the case of T-47D cells, all treatments significantly downregulated the expression of this gene (Figure 5b).

### 2.4. The Treatment with 4-OH-TMX or AM Differentially Modified mRNA Expression of Genes Involved in ER+ Signaling in Breast Cancer Cells

**Figure 6:**
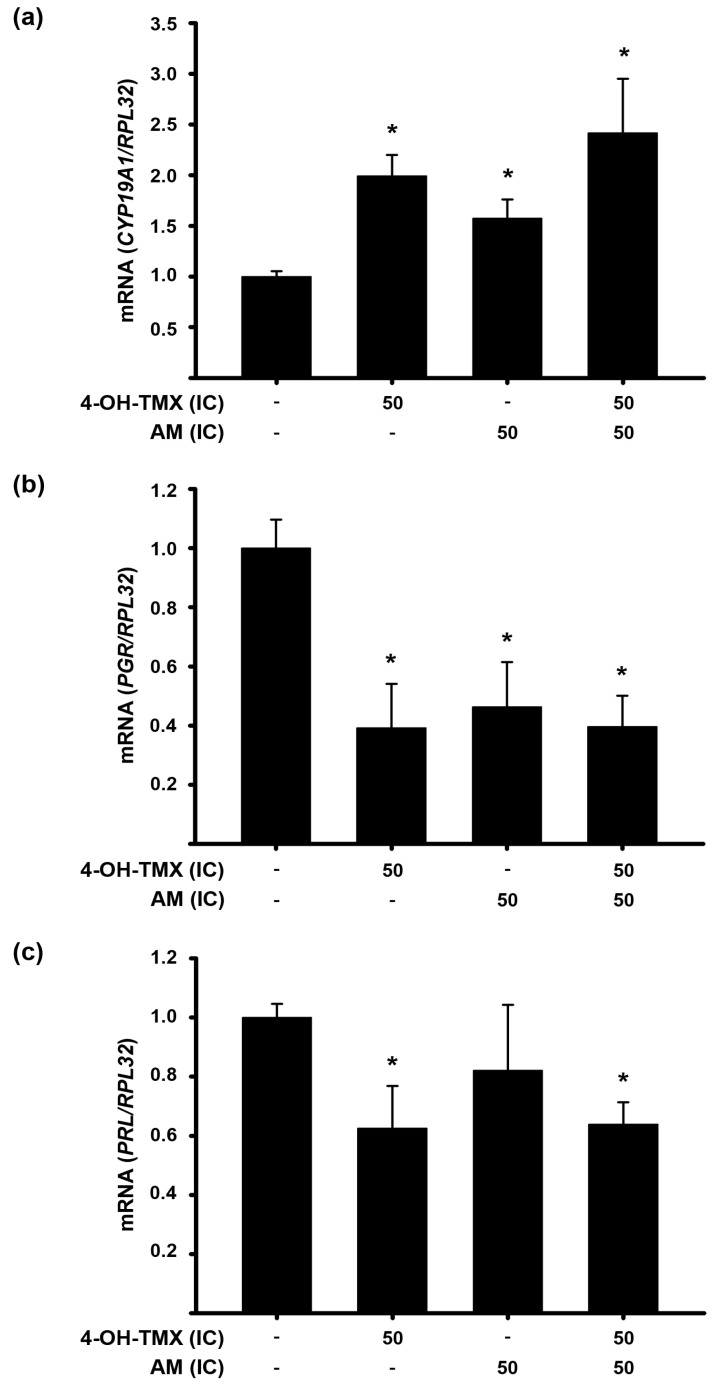
Effect 4-OH-TMX alone and combined with AM on *CYP19A1*, *PGR,* and *PRL* expression in MCF-7 cells. The effect of the inhibitory concentrations at 50% (IC_50_) of α-mangostin (AM, IC_50_ = 3.53 µM) and 4-hydroxy-tamoxifen (4-OH-TMX, IC_50_ = 2.44 µM) was evaluated upon the gene expression of (**a**) *CYP19A1*, (**b**) progesterone receptor (*PGR*), and (**c**) prolactin (*PRL*), which are involved in the synthesis of estrogens and ER signaling. The results are shown as the mean ± SEM of relative gene expression after normalizing against the ribosomal protein (*RPL32*) used as a housekeeping gene. The data from the treatments were normalized to the vehicle, to which the value of 1 was arbitrarily given, and are represented by the first bars of each graph. * *p* ˂ 0.05 vs. vehicle, *n* ≥ 4 independent experiments.

Next, we analyzed the effect of individual and combined treatments on the expression of the cytochrome P450 family 19 subfamily A member 1 (*CYP19A1*) gene, which encodes the aromatase enzyme responsible for converting androgens into estrogens. Inhibiting this enzyme can reduce estrogen production to nearly undetectable levels. Thus, we aimed to examine the effect of the treatments on *CYP19A1* gene expression in MCF-7 cells. Surprisingly, our findings revealed that 4-OH-TMX and AM alone and combined significantly increased *CYP19A1* gene expression (Figure 6a). This could potentially be attributed to a compensatory mechanism employed by the cells in response to reduced estrogen activity. To further investigate this possibility, we evaluated the effect of these compounds, alone and in combination, on the expression of the estrogen receptor 1 gene (*ESR1*) and two estrogen-regulated genes, namely, prolactin (*PRL*) and progesterone receptor (*PGR*). In our investigation, *ESR1* gene expression remained unchanged in the presence of 3.53 µM AM. In terms of the *PGR* gene’s response (Figure 6b), both 4-OH-TMX and AM significantly reduced its expression, with no additional changes observed when they were combined. Regarding *PRL* gene, its expression was significantly decreased only by 4-OH-TMX and combining it with AM did not result in any further changes (Figure 6c).

### 2.5. AM Decreases the 4-OH-TMX-Dependent Expression Upregulation of Genes Involved in Cell Proliferation in Endometrium Cells

**Figure 7:**
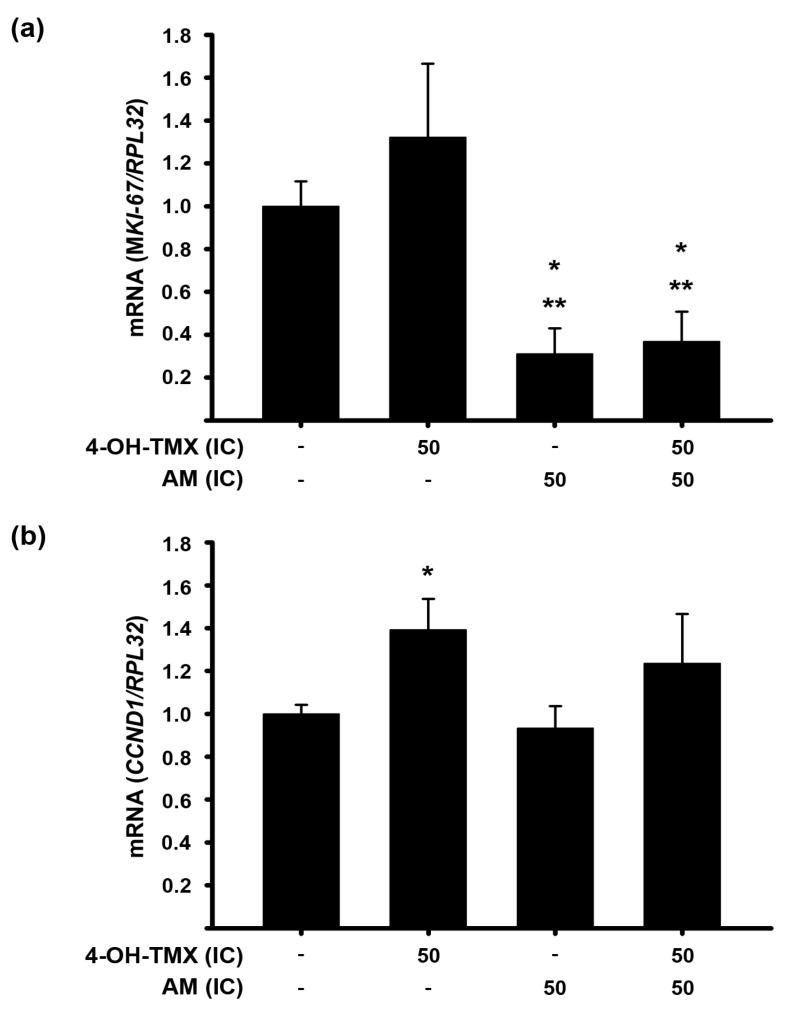
Effect of 4-OH-TMX or AM alone and combined on the gene expression of *MKI67* and *CCND1* in N30 cells. The concentrations required to inhibit MCF-7 cell proliferation in 50% (IC_50_) by α-mangostin (AM, IC_50_ = 3.53 µM) and 4-hydroxy-tamoxifen (4-OH-TMX, IC_50_ = 2.44 µM) were used as a reference to assess their impact on the gene expression of (**a**) *MKI-67* and (**b**) *CCND1* in N30 cells. The results are the mean ± SEM of relative gene expression after normalizing against the *RPL32* gene used as a housekeeping gene. The normalized values of the vehicle-treated cells are represented by the first bars of each graph. * *p* ˂ 0.05 vs. vehicle and ** *p* ˂ 0.05 vs. 4-OH-TMX; *n* ≥ 5 independent experiments with three replicates each one.

Considering the potential adverse impact of tamoxifen on the endometrium [7], alongside the anticarcinogenic effects of AM [10], we evaluated the effects of these compounds on the gene expression of *MKI-67*, which encodes the proliferation marker KI-67, as well as *CCND1* in immortalized human endometrial N30 cells. As shown in Figure 6, while 4-OH-TMX slightly increased *MKI-67* expression, AM significantly decreased it. Interestingly, when both compounds were combined, the effect of the AM prevailed (Figure 7a). Regarding *CCND1* expression, 4-OH-TMX significantly increased it; however, when it was combined with AM, this effect was prevented (Figure 7b).

## 3. Discussion

Tamoxifen, a well-established pharmacological treatment for ER+ breast cancer, has proven highly effective in reducing recurrence rates and improving disease-free survival [25]. However, its long-term usage is associated with unwanted side effects, including an elevated incidence of endometrial carcinoma [26,27]. In this in vitro study, we sought to explore the potential utility of the phytochemical AM as a co-adjuvant to conventional hormonal therapy, specifically by examining the pharmacological interaction between AM and 4-OH-TMX in two ER+ breast cancer cell lines, MCF-7 and T-47D, as well as in the endometrial cell line N30.

First of all, we assessed the impact of 4-OH-TMX and AM on breast cancer cell proliferation. This step was pivotal for us to effectively proceed with combination studies. As anticipated, both compounds exhibited the ability to inhibit cellular proliferation, and this inhibition occurred in a concentration-dependent manner in both cell lines. AM was able to completely inhibit cell growth in MCF-7 cells at 7 µM, whereas it required 20 µM to achieve the same effect in T-47D cells. Therefore, AM demonstrated a stronger effect on the proliferation of MCF-7 cells compared to T-47D cells. Our findings regarding the effect of AM on MCF-7 cells are in line with existing research, particularly the study conducted by Li et. al., who also reported a concentration-dependent decrease in MCF-7 cell proliferation in response to AM. Likewise, the calculated IC_50_ value for AM determined by us in this study (3.53 µM) closely aligned with the value of 3.57 µM reported by Li et. al., [28]. Regarding 4-OH-TMX, this drug exhibited greater effectiveness in MCF-7 cells compared to T-47D cells. Interestingly, the sensitivity of T-47D cells to 4-OH-TMX was increased by AM. The most noteworthy outcome emerged when combining AM and 4-OH-TMX, as most combination schemes exhibited a synergistic effect. Indeed, the IC_50_:IC_50_ combination for MCF-7 and T-47D yielded combination index values as low as 0.037 and 0.69, respectively.

Moreover, the combined treatment allowed for a significant dose reduction for each compound, particularly when synergism was most pronounced. The optimal dose reduction was achieved by combining the IC_50_ of AM with the IC_50_ of 4-OH-TMX. In MCF-7 cells, this combination resulted in 32-fold and 156-fold reduction for AM and 4-OH-TMX, respectively. In T-47D cells, 4-OH-TMX could be reduced by 113-fold when combined with AM. These results bear important clinical implications, as reducing compound doses can alleviate toxicity and resistance often associated with therapeutic applications.

Therefore, the benefits of combining 4-OH-TMX with AM can be distinguished. Briefly, when combined at low concentrations, a significant pharmacological effect comparable to that achieved with higher doses of 4-OH-TMX alone can be reached in vitro, thus allowing us to consider a dose reduction in 4-OH-TMX in vivo, as well as the likelihood of developing adverse effects.

To gain a mechanistic insight into the drugs’ combination synergism to inhibit breast cancer cell proliferation, we assessed the expression of cancer progression-related genes under individual and combined treatments. Our findings indicated that AM alone and in combination effectively inhibited the expression of *CCND1* in both cell lines tested. Similar results were achieved with 4-OH-TMX, but only in T-47D cells. This aligns with previous reports on AM, which indicate that its antiproliferative effects are linked to the inhibition of cyclin D1 expression, leading to cell cycle arrest in the G1 phase [16,29,30]. Cyclin D1 interacts directly with the ER, influencing gene transcription even in the absence of estrogen [31]. Considering that a significant number of ER+ breast tumors initially respond to tamoxifen therapy but eventually develop resistance through various mechanism [32,33], including the overexpression of cyclin D1 [34,35,36], our findings carry significant clinical relevance. A reduction in *CCND1* expression could inhibit the transcription of genes linked to cell proliferation, a potential consequence of combining 4-OH-TMX with AM.

On the other hand, it is known that inhibiting the EAG1 potassium channel suppresses the proliferation of breast cancer cells, arrests cell cycle progression in the G1 phase [37,38], and decreases cyclin D1 expression [39]. In this study, our data supported this relationship between EAG1 and cyclin D1, as we observed similar effects on *KCNH1* and *CCND1* expression. In MCF-7 and T-47D cell lines, we observed a significant reduction in *KCNH1* gene expression levels with AM, similar to findings reported in cervical cancer [20].

Interestingly, a noteworthy finding was that 4-OH-TMX can also suppress *KCNH1* gene expression in breast cancer cells. To the best of our knowledge, this is a novel discovery which could represent an additional mechanism by which 4-OH-TMX exerts its antineoplastic effects in estradiol-dependent ER+ breast cancer. This effect was only observed in MCF-7 cells, potentially due to the increased efficacy of the antihormonal agent in this specific cell line. Consequently, this hints at the possibility of a treatment strategy combining AM and 4-OH-TMX to inhibit the expression of the oncogenic markers *KCNH1* and *CCND1*.

Survivin protein is frequently overexpressed in many tumors, including breast cancer, where high survivin expression has been correlated with poor overall survival, suggesting its potential as a prognostic marker [22,40]. Furthermore, there is a growing consensus within the scientific community regarding the pivotal role of survivin in conferring resistance to antineoplastic drugs. Some cancer prevention compounds may function by suppressing survivin expression, while its overexpression has been associated with resistance to various antineoplastic drugs [24]. Previously, it has been reported that one of the mechanisms associated with tamoxifen-induced apoptosis resistance involves the overexpression of the anti-apoptotic molecule survivin, while its inhibition enhances tamoxifen-induced apoptosis [41]. Interestingly, in MCF-7 cells, while 4-OH-TMX and AM had no effect per se on *BIRC5* gene expression, when combined, a significant inhibition was achieved. This suggests that the combination is able to inhibit *BIRC5* gene expression, thereby enhancing the sensitivity of cells to treatment and possibly leading to better treatment outcomes. Regarding T-47D cells, all treatments inhibited survivin gene expression. This outcome could potentially lead to the development of more effective therapeutic strategies in cases where targeting survivin is essential. While these findings are promising, further research is indispensable to comprehensively grasp the implications of these results and determine their clinical applicability.

On the other hand, previous studies have described that some of the multiple mechanism through which AM decreases cancer cell proliferation involve the reduction in ER expression [42] and the inhibition of CYP19A1 activity [43] and ER antagonism [44]. Given this connection with the mechanism of action of antihormonal treatment, we were interested in evaluating the combined effects of 4-OH-TMX and AM on ER expression and signaling. As expected, 4-OH-TMX reduced the expression of estrogen-regulated genes, such as *PGR* and *PRL*. Our results related to AM suggest that this phytochemical interferes with ER signaling. This supposition is based on our observations showing a decrease in *PGR* gene expression when AM is present. The combined treatment of 4-OH-TMX with AM did not result in additional inhibition of *PGR* and *PRL* gene expression beyond what was observed with each compound alone. Future studies are required to evaluate the modifications of cyclin D1, PR, and PRL proteins by the treatments. In addition, we observed increased aromatase gene expression, which we speculate could be attributed to a cellular compensatory mechanism. This mechanism may boost protein expression in response to an estrogen shortage, possibly as an adaptive response to synthesize more estradiol; however, further evaluations of CYP19A1 protein expression and enzymatic activity are required, which are some limitations of our study. Finally, we did not observe any change in *ESR1* gene expression, possibly attributed to the concentrations of AM used in our study in contrast to higher concentrations used in previous studies [42,43].

Furthermore, we looked into the interaction of AM and 4-OH-TMX on the gene expression of *CCND1* and *MKI-67* in stromal endometrial cells, since several studies have proposed these genes as potential biomarkers for endometrial cancer development [45,46,47,48]. Interestingly, we found that the expression of the *MKI-67* gene was significantly reduced by AM and persisted when combined with 4-OH-TMX, supporting the negative effect of the phytochemical upon cell proliferation. Moreover, it is known that endometrial carcinoma cells treated with tamoxifen increase cyclin D1 expression [49,50]. In our study, this outcome was replicated by the effect of 4-OH-TMX on *CCND1* expression in N30 stromal cells, an effect that was reversed by the combination with AM to the point that it was not significantly different from the control. It is important to note that while 4-OH-TMX reduced *CCND1* expression in breast cancer cells, it actually increased its expression in endometrial cells. These observations suggest a potential protective effect of AM against the proliferative side effects of tamoxifen on endometrium.

Understanding the impact of tamoxifen on the endometrium is crucial, given its significant influence on endometrial cancer risk. However, considering the effectiveness of tamoxifen in treating breast cancer, it becomes imperative to identify compounds that can safeguard against unwanted effects.

Another recognized undesirable side effect of tamoxifen is the risk of proarrhythmic effects, such as QT-interval prolongation. This adverse effect is thought to be due to the blocking of potassium channels that regulate repolarizing currents (Ikr) in cardiomyocytes, which includes the human ether-a-go-go-related gene (HERG), a voltage-gated potassium channel from the same family as EAG1 [51,52,53,54]. Interestingly, AM has shown protective effects against cardiotoxicity induced by anticancer drugs in vivo [55]. This underscores another benefit of combining tamoxifen with AM. However, whether AM decreases the expression or activity of HERG potassium channels remains an active area of research.

In summary, the relevance of combining AM and 4-OH-TMX is multifaceted. First and foremost, the synergism observed between these compounds upon the inhibition of breast cancer cell proliferation holds excellent promise. Moreover, this synergistic interaction offers the advantage of reducing the dosage of 4-OH-TMX, potentially mitigating its adverse effects, while preserving its therapeutic efficacy. Furthermore, the combination appears to attenuate the oncogenic impact of 4-OH-TMX on the endometrium. Finally, the reported cardioprotective effects of AM could help mitigate the known tamoxifen-associated acute electrical disturbances in the myocardium, warranting further research. Overall, our findings may encourage further studies, including animal models and clinical trials, but also offer a promising avenue for advancing therapeutic strategies for patients with ER+ breast cancer.

## 4. Materials and Methods

### 4.1. Reagents

The following reagents were purchased from Sigma-Aldrich (St. Louis, MO, USA): 4-OH.TMX, AM, SRB, and trichloroacetic acid (TCA). DMSO was obtained from the American Type Culture Collection, ATCC (Manassas, VA, USA). Trizol reagent was purchased from Life Technologies (Carlsbad, CA, USA). The Maxima First Strand cDNA synthesis kit was from Thermo Fisher Scientific (Whaltham, MA, USA). The Light Cycler 480 probe Master and hydrolysis probes were purchased from Roche (Roche, Germany). Cell culture media were obtained from Life Technologies (Grand Island, NY, USA) and the fetal bovine serum (FBS) was from Gibco (Dublin, Ireland).

### 4.2. Cell Lines

The established human ER+ breast cancer cell lines MCF-7 and T-47D were purchased from the ATCC. The T-47D cells were cultured in RPMI 1640 medium and the MCF-7 cells in DMEM high-glucose medium, both supplemented with 100 U/mL of penicillin, 100 µg/mL of streptomycin, and 10% heat-inactivated FBS. The MCF-7 medium was supplemented with estradiol at a final concentration of 1 × 10^−9^ M. The N30 cell line (donated by Dr. Robert Taylor from the Obstetric and Gynecological Department, Wake Forest School of Medicine Salem, NC, USA) was derived from a biopsy of normal endometrium [56]. All experimental procedures and the culture of N30 cells were performed in DMEM-F12 medium supplemented with 100 units/mL penicillin plus 100 µg/mL streptomycin and 10% charcoal-stripped heat-inactivated FBS under standard cell culture conditions.

### 4.3. Proliferation Studies

Cells were seeded in 96-well plates (1000–2000 cells/well), and after 24 h, they were incubated by sextuplicate in the presence of different concentrations of 4-OH-TMX (1 × 10^−11^ M–1 × 10^−5^ M), AM (1.0 μM–20.0 µM), or their respective vehicles at 0.1% (DMSO for AM and ethanol for 4-OH-TMX) for 6 days. Afterward, cell proliferation was evaluated by the SRB colorimetric assay, a bright pink aminoxanthene dye that binds electrostatically to the basic amino acids of proteins under acidic conditions, providing the index of cellular protein content. Briefly, the cells were fixed with ice-cold TCA at 4 °C for 1 h and air-dried; then, the SRB (dissolved in acetic acid at 0.057%) was added to each well and incubated at room temperature for 1 h. The unbound dye was removed with three washes of acetic acid (1% *v*/*v*) and the protein-bound dye was extracted from viable cells with an alkaline solution (10 mM Tris base, pH 10.5) and shook [57]. The absorbance was read at 492 nm in a microplate reader (Synergy HT Multi-Mode Microplate Reader, BioTek, VT, USA). The concentration–response curves were generated by measuring the biological response to a range of concentrations of the compounds. Then, IC_20_ and IC_50_ values were calculated using the dose–response fitting function in the scientific graphing software Origin 9.0 (OriginLab Corporation, Northampton, MA, USA). To provide more detail, the dose–response fitting function in Origin 9.0 employs a four-parameter logistic model. This model is a standard method for analyzing dose–response data in pharmacological studies. It generates a best fit curve for the experimental data and allows for the accurate determination of the IC_50_ value, which represents the concentration of a compound required to inhibit a biological process by 50%, between the maximum and minimum effects on the concentration–response curve [58]. For combination studies, the IC_20_ and IC_50_ of 4-OH-TMX and AM alone and combined (4-OH-TMX:AM—IC_20_:IC_20_, IC_50_:IC_20_, IC_20_:IC_50_, and IC_50_:IC_50_) were used, as well as their respective vehicles.

### 4.4. Combination Index and Dose Reduction Index Determination

The pharmacological interaction between 4-OH-TMX and AM was determined by calculating the combination index and DRI, as previously reported [20]. Combination index values less than one, equal to one, or greater than one indicate synergism, an additive effect, or antagonism, respectively. Additionally, synergism is classified as slight (0.85–0.90), moderate (0.7–0.85), synergistic (0.3–0.7), strong (0.1–0.3), and very strong (<0.1), while antagonism is subdivided into slight (1.10–1.20), moderate (1.20–1.45), antagonistic (1.45–3.3), and very strong (>10) [20]. Regarding the DRI, values ˂1, =1, or >1 indicate unfavorable dose reduction, no dose reduction, or favorable dose reduction, respectively [18].

### 4.5. PCR Amplification

The effects of AM and/or 4-OH-TMX on the mRNA expression of genes involved in proliferation, oncogenesis, cell cycle progression, and ER signaling were studied by extracting total RNA from 24h-treated cells using Trizol reagent. The concentration of RNA was estimated spectrophotometrically at 260/280 nm, and 2 µg of RNA was reverse transcribed using the Maxima First Strand cDNA Synthesis kit. *RPL32* gene expression was used as a housekeeping gene. Primers sequences and universal probe library numbers are denoted in Table 3. Real-time PCR amplifications were carried out on a LightCycler^®^ 480 Instrument (Roche), according to the following protocol: activation of Taq DNA polymerase and DNA denaturation at 95 °C for 10 min, proceeded by 45 amplification cycles of 10 s at 95 °C, 30 s at 60 °C, and 1 s at 72 °C.

### 4.6. Statistical Analysis

Statistical differences were determined by one-way ANOVA followed by appropriate post-hoc tests for multiple comparisons. Comparisons between two treatments were analyzed by Student’s *t*-test using specialized software (SigmaStat 3.5, Jandel Scientific, CA, USA). Differences were considered statistically significant at *p* ˂ 0.05.

## 5. Conclusions

This study provides insights into the pharmacological interaction between α-mangostin and the hormonal therapy commonly used for breast cancer treatment. The synergistic effect of combining the active metabolite of tamoxifen with AM in breast cancer cells is beneficial and noteworthy. Importantly, in endometrial cells, AM inhibited the tamoxifen-induced increase in the gene expression of the cell cycle progression marker *CCND1.* Moreover, it significantly reduced the gene expression of the proliferation marker *MKI67*, an effect that persisted when combined with 4-OH-TMX. Therefore, the combined administration of tamoxifen and AM in the clinical use could be a promising therapeutic option.

## Figures and Tables

**Table 1:** Cell proliferation IC_20_ and IC_50_ values of AM and 4-OH-TMX in breast cancer cell lines.

Cell Line	AM (µM)		4-OH-TMX (µM)	
	IC_20_	IC_50_	IC_20_	IC_50_
MCF-7	2.35 ± 0.28	3.53 ± 0.23	0.73 ± 0.58	2.44 ± 2.35
T-47D	4.60 ± 0.22	7.15 ± 0.16	0.0094 ± 0.0072	0.1584 ± 0.082

Inhibitory concentrations at 20% (IC_20_) and 50% (IC_50_) of α-mangostin (AM) and 4-hydroxy-tamoxifen (4-OH-TMX) in breast cancer cells. The results are the mean ± SEM of at least four independent experiments.

**Table 2:** Dose reduction index (DRI) of 4-OH-tamoxifen and α-mangostin combined in ER+ breast cancer cell lines.

Cell Line	Combination Schemes	DRI (Folds)	
	4-OH-TMX:AM	4-OH-TMX	AM
MCF-7	IC_20_:IC_20_	1.38	0.82
	IC_50_:IC_20_	3.44	3.52
	IC_20_:IC_50_	15.28	2.86
	IC_50_:IC_50_	156.18	32.63
T-47D	IC_20_:IC_20_	7.55	1.36
	IC_50_:IC_20_	0.43	1.37
	IC_20_:IC_50_	85.16	1.29
	IC_50_:IC_50_	113.67	1.46

DRI was calculated for the combination of 4-OH-tamoxifen (4-OH-TMX) and α-mangostin (AM) at the inhibitory concentrations of 20% (IC_20_) and/or 50% (IC_50_). For further details on inhibitory concentrations, refer to Table 1. A DRI value >1, =1, and <1 indicates a favorable, no effect, or negative dose reduction, respectively.

**Table 3:** Primers and probes.

Gen	Accession Number	Upper Primer	Lower Primer	Probe Number *
*KCNH1*	AF078741.1	cctggaggtgatccaagatg	ccaaacacgtctccttttcc	49
*MKI67*	X65550.1	ggtgtgcagaaaatccaaga	actgtccctatgacttcttctggttg	63
*CCND1*	NM_053056.2	gaagatcgtcgccacctg	gacctcctcctcgcacttct	67
*BIRC5*	NM_001012271.2	gcccagtgtttcttctgctt	aaccggacgaatgcttttta	11
*ESR1*	X03635.1	ccttcttcaagagaagtattcaagg	gtttttatcaatggtgcactgg	83
*CYP19A1*	NM_00103.2	gaattcatgcgagtctggatct	tcattatgtggaacatacttgagga	55
*PGR*	NM_001271162	tcaagcttcaagttagccaaga	gacttcgtagcccttccaaa	6
*PRL*	NM_000948.2	aaaggatcgccatggaaag	gcacaggagcaggtttgac	18
*RPL32*	NM_000994.3	gaagttcctggtccacaacg	gagcgatctcggcacagta	17

* From the universal probe library (Roche).

## Data Availability

Data is contained within the article.
